# Takotsubo cardiomyopathy prevalence and associated factors in patients presenting with a clinical picture of acute myocardial infarction in Palestine

**DOI:** 10.1186/s43044-023-00399-6

**Published:** 2023-08-14

**Authors:** Sameer Mtour, Lila H. Abu-Hilal, Duha I. Barghouthi, Yumna Njoum, Farah Jabbarin, Bilal Adwan, Ibrahim Abu Asbeh, Ali Mtour, Isaac Alsallamin

**Affiliations:** 1 Cardiology Department, Al-Makassed Hospital, Jerusalem, Palestine; 2 Faculty of Medicine, Al-Quds University, Jerusalem, Palestine

**Keywords:** Takotsubo cardiomyopathy, Broken heart syndrome, Stress-induced cardiomyopathy, Prevalence, ACS, Palestine

## Abstract

**Background:**

Takotsubo cardiomyopathy (TC) is a transient cardiac syndrome that manifests with symptoms resembling acute myocardial infarction (MI). It is characterized by temporary wall-motion abnormalities predominantly affecting the apical and mid-portions of the left ventricle, despite the absence of significant obstructive coronary disease. TC poses diagnostic challenges due to its resemblance to ST-segment elevation myocardial infarction.

Our study aimed to determine the prevalence of TC and identify the factors associated with its occurrence in patients presenting with acute MI in Palestine.

**Results:**

A retrospective analysis was conducted on a cohort of patients diagnosed with TC at Al-Makassed hospital. Women accounted for 90.7% of TC cases (95% CI 88.2–93.2%). The mean age of affected individuals ranged from 62 to 76 years. The most common presenting symptoms were chest pain (83.4%, 95% CI 80.0–86.7%) and dyspnea (20.4%, 95% CI 16.3–24.5%), often following an emotionally or physically stressful event. Electrocardiography (ECG) on admission indicated ST-segment elevations in 71.1% of cases (95% CI 67.2–75.1%), accompanied by mild elevations of Troponins in 85.0% of cases (95% CI 80.8–89.1%). Despite the initial severity, left ventricular ejection fraction (LVEF) improved from 20–49.9 to 59–76% within a mean time of 7–37 days. The in-hospital mortality rate was 1.7% (95% CI 0.5–2.8%), with complete recovery observed in 95.9% of cases (95% CI 93.8–98.1%) and rare recurrence. The underlying etiology is believed to involve exaggerated sympathetic stimulation.

**Conclusions:**

TC should be considered as a significant differential diagnosis in acute coronary syndrome (ACS) cases, particularly among postmenopausal women with a preceding stressful event. Our study provides insights into the prevalence and characteristics of TC in the Palestinian population. While stress has been recognized as a potential trigger for TC, further research is needed to explore if there are specific associations between occupation and other unique stressors in the Palestinian context and the prevalence of TC. The study’s results can raise awareness among healthcare professionals in Palestine about the prevalence and characteristics of TC in their patient population.

## Background

Transient left ventricular apical ballooning syndrome or broken heart syndrome is an acute cardiac syndrome mimicking ST-segment elevation myocardial infarction characterized by transient wall-motion abnormalities involving apical and mid-portions of the left ventricle in the absence of significant obstructive coronary disease [1]. Since its initial characterization by the Japanese scientist Sato in 1990, TC has gained recognition as a distinct clinical entity [2].

The term “Takotsubo” derives from the Japanese word for octopus trap, reflecting the unique appearance of the left ventricle resembling an inflated octopus trap [3].

Typically, TC predominantly affects postmenopausal women over the age of 50 who have myocardial infarction-like complaints, such as sudden onset chest discomfort, syncope, or dyspnea, which is triggered by emotional stress and is defined by the development of apical ballooning on echocardiography [2]. As a differential diagnosis, concomitant coronary artery disease has been recorded in Takotsubo patients, with an incidence ranging from 10 to 29%. As a result, individuals with Takotsubo and obstructive coronary artery disease are frequently mistaken as having a typical ACS, and distinction can be difficult. Furthermore, there are certain instances in which doctors must distinguish Takotsubo from myocarditis, myocardial infarction, and nonobstructive coronary arteries [2]. Furthermore, Takotsubo can appear at a younger age, and patients are frequently hospitalized with signs of abrupt heart failure and cardiogenic shock, pulmonary edema, or stroke [2]. Many disorders have been related to TC, including sympathetic overstimulation, microvascular and myocardial tissue metabolic abnormalities, and coronary artery vasospasm [1]. Improved clinical awareness and broad availability to coronary angiography have resulted in an increase in diagnosis in recent years. Takotsubo has been recorded in 1–2% of all “troponin-positive” suspected ACS presentations and almost 6% of all women presenting with suspected STEMI who receive urgent angiography [3].

ECG changes in TC occur in distinct phases. ST-segment deviation that occurs during the first several hours of the beginning of symptoms is stage 1. Stage 2 includes progressive deep T-wave inversion and QTc prolongation, which peak between days one and three and two and six. When the QTc is adjusted for gender, however, female patients with acute MI have similar QTcs. These T-wave inversions frequently occur over the precordial (V1 through V6), bipolar (I, II), and lateral limb (aVL) leads and are associated with myocardial edema that may last after the cardiac contraction has returned [4].

Torsade de pointes and other ventricular tachycardias can happen in the first 24 to 48 h. Takotsubo syndrome is known to be associated with prolonged QTc segments in ventricular arrhythmias. However, it seems uncommon that ventricular arrhythmias in the hyperacute stage of Takotsubo syndrome are connected to QTc lengthening. It is also possible for people to initially be diagnosed with ventricular arrhythmia and then later develop Takotsubo syndrome, making it exceedingly challenging to determine which happened first. In stage 3, T-wave and QTc alterations gradually disappear over the coming weeks or months. Contrary to the simultaneous recovery of contractile function and the ECG found following restoration of blood flow in the heart, the normalization of myocardial contractile function occurred before persistent electrocardiographic anomalies. In striking contrast to the simultaneous recovery of contractile function and the ECG seen following restoration of blood flow in ischemic myocardial stunning, normalization of myocardial contractile function occurs prior to long-lasting electrocardiographic anomalies. These ideas would argue the idea that traditional myocardial ischemia contributed to the pathophysiology of Takotsubo syndrome, together with the relative lack of isolated ST depression on the presentation ECG [4].

Given the unique characteristics and challenges associated with TC, this study aims to determine the prevalence of TC and identify factors associated with its occurrence in patients presenting with acute myocardial infarction in Palestine. This research is essential to improve understanding, raise awareness, and provide valuable insights for healthcare professionals in Palestine regarding the prevalence and characteristics of TC in their patient population.

## Methods

This retrospective cohort study was conducted at our hospital to investigate the prevalence of TC in patients with STEMI who presented with a final diagnosis of apical ballooning. The study period spanned from 2018 to 2022.

A total of 145 women with an initial presentation of anterior-STEMI (A-STEMI) were hospitalized and underwent urgent coronary angiography and echocardiography. Patients with evidence of plaque rupture or intracoronary thrombus formation on angiography were excluded from the study.

Data for this study were collected retrospectively, information regarding demographic and clinical characteristics, including troponin levels, was extracted and analyzed.

Descriptive statistics, including proportions, means, standard deviations, medians, and interquartile ranges, were used to summarize categorical variables. The independent sample t test was employed to compare categorical variables. Multivariate analysis was performed using logistic regression models, considering potential mediators or confounding factors. The statistical significance level was set at *p* < 0.05, and all statistical tests were two-sided. Statistical analyses were conducted using the Statistical Package for the Social Sciences (SPSS) version 23.0.

## Results

In our study, we collected data from all patients who were initially diagnosed with A-STEMI and later diagnosed with apical ballooning.

Out of the total A-STEMI cases (699), a small proportion (6%, *n* = 44) were diagnosed with TC.

Among the female patients, 42 out of 145 (29%) were diagnosed with TC, highlighting a significant association between gender and the development of TC in the A-STEMI population (*p* < 0.00001). In contrast, the prevalence of TC in male A-STEMI patients was notably lower, with only 2 out of 554 (0.4%) cases being identified as TC (Table 1).

**Table 1:** Takotsubo cardiomyopathy in A-STE by gender

	ALL A-STE 699	A-STEMI (94%) 44	Takotsubo (6%) 44	*p* value
Female	145 (21%)	103 (71%)	42 (29%)	< 0.00001
Male	554 (79%)	552 (99.6%)	2 (0.4%)	

For further analysis, we focused on the cohort of female patients who presented with A-STEMI. Among the total cohort, we identified 145 women who were hospitalized with an initial presentation of A-STEMI. Urgent coronary angiography and echocardiography were performed for all patients, allowing for a comprehensive evaluation of cardiac function and identification of additional cases of TC.

### Prevalence and characteristics of Takotsubo cardiomyopathy

**Fig. 1:**
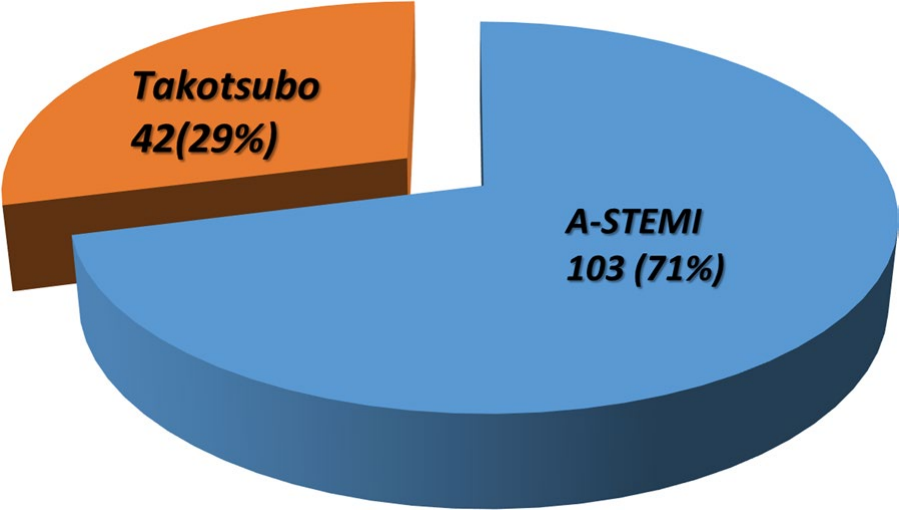
In 42 out of 145 (29%) of the women, normal coronaries were found and on echocardiography apical ballooning was present

Interestingly, in 42 out of 145 (29%) women, normal coronaries were observed during the angiography, while echocardiography revealed the presence of apical ballooning. Notably, none of our patients showed angiographic evidence of plaque rupture or intracoronary thrombus formation, and no patients were diagnosed with concurrent ACS and TC (Fig. 1).

We noticed that the proportion of female patients presenting with apical ballooning has increased over the years, possibly due to improved recognition and diagnostic capabilities. TC has been reported in a certain percentage of patients with suspected ACS. In our study, we found that TC affects women in 90.7% of cases (95% CI 88.2–93.2%), with an average age ranging from 62 to 76 years.

The most common symptoms included chest pain (83.4%, 95% CI 80.0–86.7%) and dyspnea (20.4%, 95% CI 16.3–24.5%), often occurring following an emotionally or physically stressful event.

### Comparison of risk factors between Takotsubo and A-STEMI patients

**Fig. 2:**
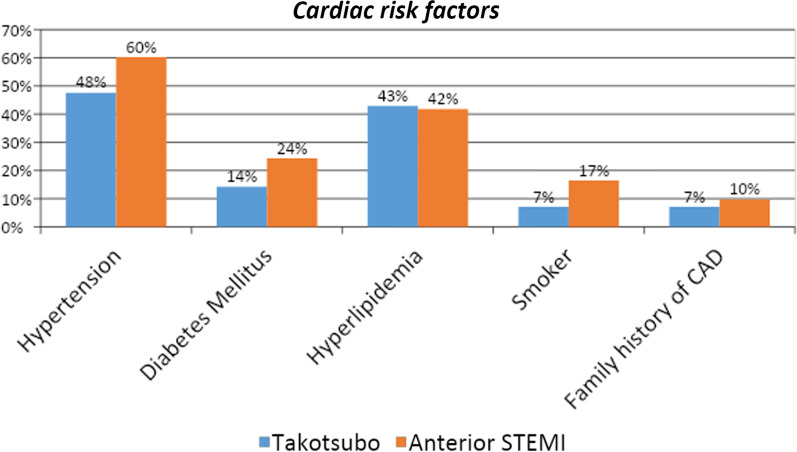
Cardiac risk factors in Takotsubo versus anterior-STEMI patients

The mean age of patients with TC was 67.4 ± 9.7 years, slightly lower than the overall average of 69.7 ± 11.5 years. Second, hypertension was present in 47.6% of TC cases compared to 56.6% in the A-STEMI group, although this difference was not statistically significant (*p* = 0.166). Similarly, diabetes mellitus was observed in 14.3% of Takotsubo cases and 24.3% of A-STEMI cases, with no significant difference between the groups (*p* = 0.183). In addition, hyperlipidemia, smoking, and family history of coronary artery disease did not show significant differences between the Takotsubo and A-STEMI groups. Lastly, the number of risk factors tended to be lower in the Takotsubo group (median 1, range 0–2) compared to the A-STEMI group (median 2, range 1–2), with a trend toward statistical significance (*p* = 0.070) (Table 2, Fig. 2).

**Table 2:** Baseline characteristics of patients with TC versus A-STEMI

	All (145)	Takotsubo (42)	A-STEMI (103)	*p* value
Age in years*	69.7 ± 11.5	67.4 ± 9.7	68.8 ± 12.2	0.519
Hypertension	82 (56.6%)	20 (47.6%)	62 (60.2%)	0.166
Diabetes mellitus	31 (21.4%)	6 (14.3%)	25 (24.3%)	0.183
Hyperlipidemia	61 (42.1%)	18 (42.9%)	43 (41.7%)	0.902
Smoker	20 (13.8%)	3 (7.1%)	17 (16.5%)	0.138
Family history of CAD	13 (9.0%)	3 (7.1%)	10 (9.7%)	0.624
Number of risk factors**	[1–2]1	[0–2]1	[1–2]2	0.07

*Mean ± SD

**Median [Interquartile range]

### Troponin levels and diagnosis

**Fig. 3:**
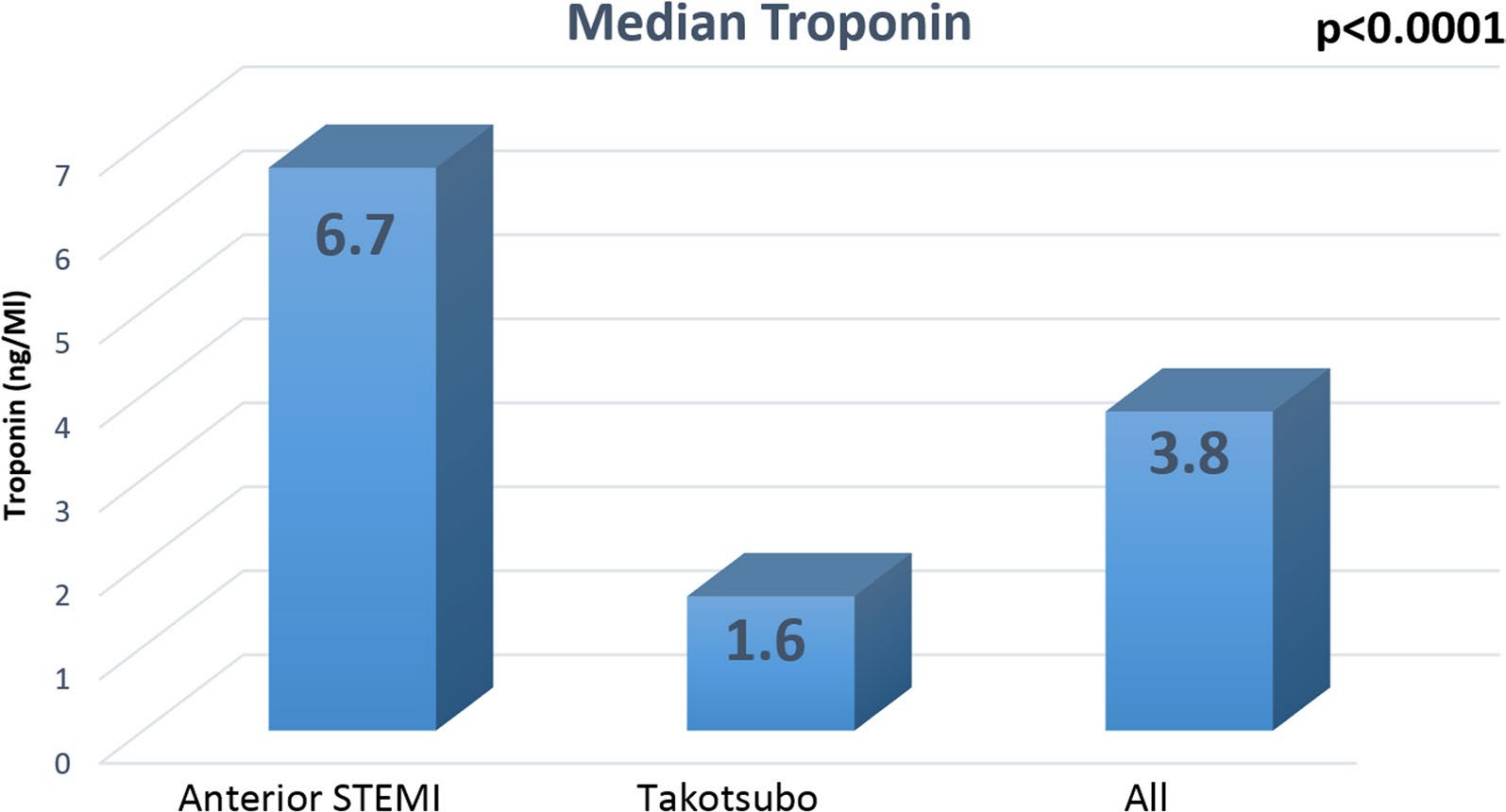
Median troponin

We also assessed the role of troponin levels in diagnosing TC and found that maximum troponin levels did not demonstrate statistical significance. The median troponin level in patients with A-STEMI was higher compared to TC patients (Fig. 3).

### Clinical course and outcomes

Electrocardiography conducted upon admission revealed ST-segment elevations in 71.1% of patients (95% CI 67.2–75.1%), accompanied by typically mild elevations of Troponins in 85.0% of cases (95% CI 80.8–89.1%). Despite the dramatic clinical presentation and the significant risks of heart failure, cardiogenic shock, and arrhythmias, we observed an improvement in left ventricular ejection fraction from 20–49.9 to 59–76% within an average time of 7–37 days.

Among the patients who underwent two echocardiogram tests during hospitalization 30 (71.5%), 17 patients showed improved left ventricular function at discharge. This improvement indicates a positive response to the treatment and management strategies employed. Furthermore, 13 out of these 30 patients had achieved normal left ventricular function at the time of discharge, indicating a complete recovery of cardiac function. The significant proportion of patients with improved and normalized left ventricular function highlights the favorable prognosis and the ability of the heart to recover following TC. This finding emphasizes the importance of close monitoring and follow-up to evaluate the progress and recovery of left ventricular function in patients with TC (Table 3).

**Table 3:** Left ventricle function follow-up

Left ventricular function	
Patient who underwent two echocardiogram tests during hospitalization	30 (71.5%)
Improved left ventricular function at discharge	17 (56.7%)
Normal left ventricular function at discharge	13 (43.3%)

The in-hospital mortality rate was found to be 1.7% (95% CI 0.5–2.8%), with a complete recovery observed in 95.9% of patients (95% CI 93.8–98.1%) and rare instances of recurrence. The underlying etiology of TC is believed to be associated with an exaggerated sympathetic stimulation.

These findings shed light on the characteristics and outcomes of TC in our patient population. By documenting the rise in female patients with apical ballooning and highlighting the clinical course, our study contributes to the growing body of knowledge regarding this unique cardiac syndrome.

## Discussion

Our study provides important insights into the prevalence and associated factors of TC in patients initially diagnosed with A-STEMI in Palestine. We observed a significant difference in the prevalence of TC between male and female patients, with a higher incidence among females (29%) compared to males (0.4%). This gender disparity aligns with findings from the GEIST registry, which also reported a higher proportion of TC cases in females [5]. However, further research is needed to better understand the underlying factors contributing to this gender discrepancy and the impact of other risk factors.

Although male sex is characterized by a distinct high-risk phenotype, including presence of comorbidities, physical triggers, and adverse outcomes requiring close in-hospital monitoring and subsequent follow-up. When matched for underlying comorbidities and triggers, men retained worse in-hospital outcome, albeit similar long-term prognosis, compared with women [5]. In contrast to the more well-known broken heart syndrome, which is linked to detrimental emotional stressors, happy heart syndrome is an uncommon form of TC that is preceded by pleasant triggers and has a higher prevalence of male patients, although very scarce research has been done regarding this phenomena and requires wide-based research [6].

Postmenopausal women showed a higher prevalence of TC, as demonstrated in our study where the mean age of patients with TC was 67.4 ± 9.7 years, consistent with previous reports [5, 7–9]. It has been postulated that underlying predisposing factors more prevalent among postmenopausal women may facilitate TC by lowering the threshold for adrenergic stimulation required to initiate the syndrome. Conversely, higher estrogen levels in premenopausal women may exert a cardioprotective effect against the development of TC. This highlights the role of hormonal factors in the pathogenesis of TC and warrants further investigation [5, 10].

TC shares the conventional cardiovascular risk factors, similar to the other major cardiovascular diseases including hypertension, diabetes, hyperlipidemia, chronic kidney disease, gender, obesity, and age [11].

Regarding listed risk factors, our study found no significant differences in the prevalence of hypertension and diabetes mellitus between the TC and A-STEMI groups. Although hypertension has been reported in a substantial proportion of TC cases in previous studies, the association between hypertension and TC remains inconclusive [12, 13]. Similarly, while complications of diabetes mellitus have been reported to trigger TC in some cases, the clear association between the two conditions is not well established [11].

Furthermore, our study did not identify significant differences in the prevalence of hyperlipidemia, smoking, and family history of coronary artery disease between the TC and A-STEMI groups. These findings suggest that these risk factors may not play a major role in the development of TC, at least in our studied population.

Differentiating TC from A-STEMI is crucial, as the treatment approaches differ. Patients with STEMI undergo cardiac catheterization as a first diagnostic step, this functions along with clinical assessment, detection of elevated BNP/NT-proBNP levels, ECG performance and echocardiography as initial diagnostic tests and should be followed by the demonstration of extensive myocardial edema on cardiac magnetic resonance imaging (CMR) [14].

In our study, due to limited resources at our hospital, we followed a similar diagnostic pathway, focusing on troponin levels, without relying on BNP/NT-proBNP levels, or CMR. In our investigation, we assessed the role of troponin levels in diagnosing TC and found that maximum troponin levels did not demonstrate statistical significance between TC and A-STEMI patients. Interestingly, the median troponin level was higher in patients with A-STEMI compared to those with TC. These findings highlight the limitation of using troponin levels alone as a diagnostic marker for TC.

In terms of outcomes, TC is characterized by substantially recovered systolic function. Our study showed that 71.65% of patients with TC demonstrated improved left ventricular function as assessed by second echocardiography. The in-hospital mortality rate was low (1.7%), with a high proportion of patients (95.9%) experiencing complete recovery. Recurrence of TC was rare. However, it is important to note that noncardiovascular events can contribute to mortality in the long-term follow-up of TC patients [5].

Overall, our findings suggest that TC predominantly affects patients in their late 60 s and is more prevalent in individuals without hypertension and diabetes mellitus compared to A-STEMI patients. While further research is needed to better understand the underlying mechanisms and risk factors associated with Takotsubo cardiomyopathy, this study provides valuable insights into the prevalence and associated factors of this condition in the Palestinian population.

Our study contributes to the existing literature on Takotsubo cardiomyopathy by providing specific insights into its prevalence and associated factors in patients presenting with acute myocardial infarction in Palestine. This study adds to the current body of knowledge by reporting the prevalence of Takotsubo cardiomyopathy in this particular population and identifying the predominance of female cases. Additionally, our study sheds light on the clinical presentation, symptoms, and outcomes of patients with Takotsubo cardiomyopathy, including the improvement in left ventricular ejection fraction and low in-hospital mortality rate. By focusing on the Palestinian population, our research offers valuable regional data that can enhance the understanding of this condition and inform future studies and clinical practices in the area.

### Study limitations

Firstly, the study was conducted at a single center in Palestine, which may limit the generalizability of the findings to the broader population. The patient population at this center may not fully represent the diversity of patients with acute myocardial infarction in Palestine, potentially introducing selection bias. Secondly, the retrospective nature of the study design poses inherent limitations. The reliance on medical records and available data may lead to incomplete or missing information. In this study, one notable limitation is the lack of second echocardiography assessment in all patients. This could potentially affect the accurate diagnosis and classification of TC cases and limit the comprehensive evaluation of its clinical characteristics and outcomes.

Lastly, the limited sample size and single-center nature of the study may restrict statistical power and precision, potentially limiting the ability to detect significant associations or draw firm conclusions. A larger, multicenter study with a prospective design would provide more robust and generalizable results. Overall, while this retrospective single-center study provides valuable insights into the prevalence and associated factors of TC in myocardial infarction patients in Palestine, the results should be interpreted with caution due to the inherent limitations.

## Conclusions

In summary, this retrospective study conducted in Palestine revealed a remarkable gender disparity in the prevalence of TC among acute myocardial infarction patients. The findings showed a high prevalence of TC among women with A-STEMI, reaching 29%, while men had a significantly lower prevalence at 0.4%. The study highlights the need for further research to investigate the underlying factors contributing to this gender disparity, including potential influences from the occupation and other contextual factors. Understanding the causes and mechanisms behind this disparity would provide valuable insights for developing targeted preventive and therapeutic strategies in Palestine.

## Data Availability

The datasets used and/or analyzed during the current study are available from the corresponding author on reasonable request.

## Abbreviations

TC: Takotsubo cardiomyopathy
MI: Myocardial infarction
STEMI: ST-segment elevation myocardial infarction
ECG: Electrocardiography
LVEF: Left ventricular ejection fraction
ACS: Acute coronary syndrome
A-STE: Anterior ST-segment elevation
A-STEMI: Anterior ST-segment elevation myocardial infarction
SPSS: Statistical Package for the Social Sciences
GEIST: The GErman Italian Spanish Takotsubo
BNP: Brain natriuretic peptide
Nt-proBNP: N-terminal prohormone of brain natriuretic peptide
CMR: Cardiac magnetic resonance imaging
